# Genome-wide analysis of GRAS transcription factor gene family in *Gossypium hirsutum* L.

**DOI:** 10.1186/s12864-018-4722-x

**Published:** 2018-05-09

**Authors:** Bin Zhang, J. Liu, Zhao E. Yang, Er Y. Chen, Chao J. Zhang, Xue Y. Zhang, Fu G. Li

**Affiliations:** 1 Research Base, Anyang Institute of Technology, State Key Laboratory of Cotton Biology, Anyang, 455000 China; 2 Institute of Cotton Research, Chinese Academy of Agricultural Sciences, Anyang, 455000 China

**Keywords:** *Gossypium hirsutum* L., GRAS family, Abiotic stress, Phylogeny, Duplication

## Abstract

**Background:**

Cotton is a major fiber and oil crop worldwide. Cotton production, however, is often threatened by abiotic environmental stresses. GRAS family proteins are among the most abundant transcription factors in plants and play important roles in regulating root and shoot development, which can improve plant resistance to abiotic stresses. However, few studies on the GRAS family have been conducted in cotton. Recently, the *G. hirsutum* genome sequences have been released, which provide us an opportunity to analyze the GRAS family in *G. hirsutum.*

**Results:**

In total, 150 GRAS proteins from *G. hirsutum* were identified. Phylogenetic analysis showed that these GRAS protins could be classified into 14 subfamilies including SCR, DLT, OS19, LAS, SCL4/7, OS4, OS43, DELLA, PAT1, SHR, HAM, SCL3, LISCL and G_GRAS. The gene structure and motif distribution analysis of the GRAS members in *G. hirsutum* revealed that many genes of the SHR subfamily have more than one intron, which maybe a kind of form in the evolution of plant by obtaining or losing introns. Chromosomal location and duplication analysis revealed that segment and tandem duplication maybe the reasons of the expension of the GRAS family in cotton. Gene expression analysis confirmed the expression level of GRAS members were up-regulated under different abiotic stresses, suggesting that their possible roles in response to stresses. What’s more, higher expression level in root, stem, leaf and pistil also indicated these genes may have effect on the development and breeding of cotton.

**Conclusions:**

This study firstly shows the comprehensive analysis of GRAS members in *G. hirsutum.* Our results provide important information about GRAS family and a framework for stress-resistant breeding in *G. hirsutum*.

**Electronic supplementary material:**

The online version of this article (10.1186/s12864-018-4722-x) contains supplementary material, which is available to authorized users.

## Background

Transcription factors are proteins that play important roles in plant growth, development and resistance to abiotic environmental stresses, such as cold, hot, drought and salt. In general, transcription factors function by combining with other proteins or DNA sequences to promote or inhibit gene transcription. Some of the known families of transcription factors including WRKY, MYB, MADS, ARF, AP2/EREBP, HB, SBP, bZIP and GRAS families, have been well studied [1–3]. The GRAS transcription factor family is a plant-specific gene family named after the first three functionally characterized genes, GAI [4], RGA [5], and SCR [6]. And GRAS family likely emerged first in bacteria [7]. Generally, GRAS proteins range from 400 to 700 amino acid residues [8]. According to previous reports, GRAS proteins can be divided into at least 13 subfamilies including SCR, DLT, OS19, LAS, SCL4/7, OS4, OS43, DELLA, PAT1, SHR, HAM, SCL3 and LISCL [8]. GRAS proteins contain a conserved sequence in their carboxyl terminus, which typically contains several ordered motifs including LHRI, VHIID, LHRII, PFYRE and SAW [9]. Up to now, GRAS transcription factors have been found in more and more species, such as *Arabidopsis thaliana* [9], *Oryza sativa* [9], *Castor Bean* [8], *tobacco* [10], *Populus* [11], *Chinese cabbage* [12], *Grapevine* [13], *Zea mays L*. [14] and *Nelumbo nucifera* [15].

GRAS proteins play important roles in many processes such as signal transduction, axillary shoot meristem formation, root radial patterning, stress responses [16] and meristem maintenance [17]. The genes *GAI*, *RGA*, and *RGL* of DELLA subfamily were reported as repressors of gibberellin signaling [18] . Besides, previous reports confirmed that SCR and SHR could form the SCR/SHR complex, which was related with the root radial patterning [19] . In addition, SCR also controls Arabidopsis root meristem size from the root endodermis tissue by regulating the DELLA protein RGA [19]. What’s more, SCL3 was proved as a factor mediating the elongation of root [20]. When the homolog of MOC1 in Arabidopsis, AtLAS, was knockout, forming lateral shoots during vegetative development became impossible [21]. In addition, the triple *scl6* mutants performed similar pleiotropic phenotypes of meristematic cells when overexpressing miR171 [22]. Both the *shr aba2* and *scr aba2* double mutants of *A. thaliana* suppressed the effects of stresses on root meristematic activity [23]. Thus, GRAS transcription factors play important regulatory roles in the development of roots and shoots, which are always related with resistance to abiotic stresses. And several genes reported played a positive role in plant stress responses. The Arabidopsis GRAS protein SCL14 was shown to be essential for the activation of stress-inducible promoters [24]. What’s more, NtGRAS1 in tobacco could raise the ROS level under various stresses [16]. Overexpressing *PeSCL7* of *P. euramaricana* in Arabidopsis enhanced its tolerance to drought and salt treatments [25]. And OsGRAS23 was reported as a positive factor for stress resistance [26]. Besides, Overexpression of VaPAT1, a GRAS member from *Vitis amurensis*, confers abiotic stress tolerance in Arabidopsis [27].

Cotton is one of the most important fiber and oil crops worldwide but cotton production is always threatened by abiotic stresses, such as cold, heat, drought and salinity [28]. On one hand, breeding for stress-resistant cotton is a high priority for plant biotechnology programs. As plant-specific transcription factors, GRAS members play important roles in phytohormone pathways and the crosstalk among them [29]. DELLA-like genes have possible effect on cell elongation, influencing plant hight or root elongation, which may regulate the resistance to stresses [30]. Additionally, the GRAS family was enriched in the early response to salt stress in an analysis of serial cDNA libraries [31]. Therefore, studying the GRAS protein in cotton could help us breed stress-resistant cotton. Here, we identified 150 GRAS members in *G. hirsutum*. The phylogenetic relationships, locations, structures and expression patterns of these genes were analyzed. About 37 persents of members cotaining introns were members of SHR subfamily, which maybe a kind of possible evolution pattern by obtaining or losing introns. And tandem should be the reason of the expansion of SHR family members. Ka/Ks of segmental gene pairs were lower than 1, suggesting that purified selection acted on these duplicated genes. In addition, the expression profiles of the GRAS members showed that they were differentially expressed under various stress treatments and among tissue. And we selected 15 genes up-regulated under stresses to confirm via qRT-PCR experiment, getting a similar result. Several genes could be good candidates for stress-resistant cotton breeding.

## Results

### Identification of GRAS family members

We identified a total of 150 GRAS proteins in *G. hirsutum* by a combination of methods. Additionally, 77 GRAS members in *G. arboreum*, 82 GRAS members in *G. raimondii*, 33 GRAS members in *A. thaliana* [9], 42 GRAS members in *P. patens*, 46 GRAS members in *S. moellendorffii* and 50 GRAS members in *O. sativa* [8] were identified with the same methods (Additional file 1: Table S1). The number of GRAS members in *G. hirsutum* (AD group) was about twice that in *G. arboreum* (A group) and *G. raimondii* (D group), which was consistent with their tetraploid and diploid genomes. Basic information, including gene length, subfamily, isoelectric point (pIs), and molecular weight (MWs), for the GRAS proteins in *G. hirsutum* is listed (Additional file 1: Table S1). Information on the GRAS proteins in the other species is provided (Additional file 1: Table S1). The identified GRAS encoded proteins ranging from 208 (*Gh_D04G1304*) to 1821 (*Gh_D02G0276*) amino acids, with pIs varying from 4.68 (*Gh_A09G2415*) to 8.99 (*Gh_A05G0802*) and MWs varying from 23,116.5 D (*Gh_D04G1304*) to 208,508.85 D (*Gh_D02G0276*). And, 141 members can be located on certain chromosomes, 67 members in the A group chromosomes and 74 members in D group chromosomes.

### Phylogenetic analysis of GRAS members

**Fig. 1:**
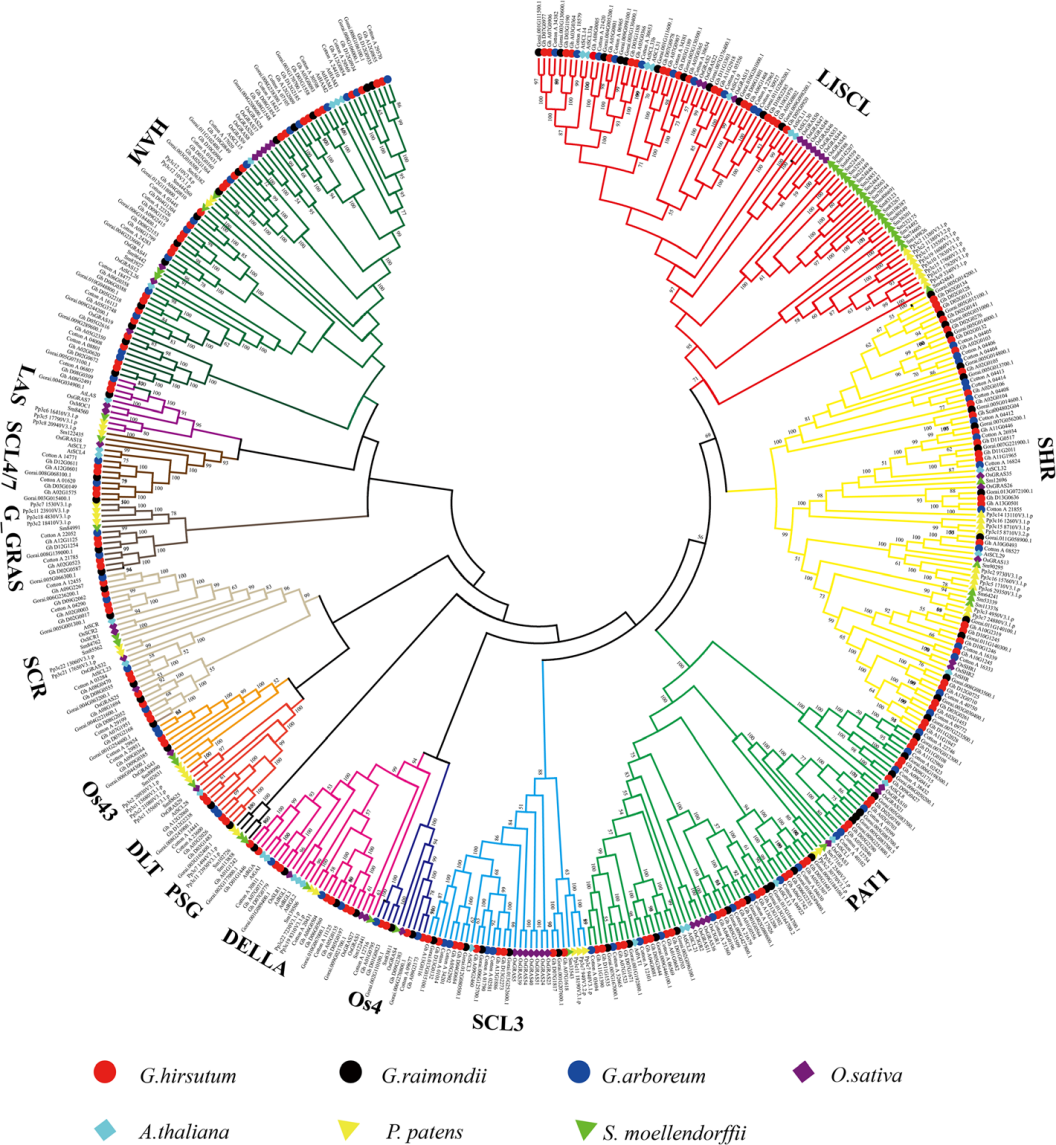
An unrooted phylogenetic tree of GRAS gene family. The multiple sequence alignment of GRAS domain sequences in *O. sativa*, *P. patens*, *S. moellendorffii*, *A. thaliana*, *G. hirsutum*, *G. arboreum* and *G. raimondii* was carried out using MUSCLE method in MEGA, and the tree was generated with neighbor-joining method. Members in the same clade with special color belong to the same subfamily

We built a NJ phylogenetic tree (Fig. 1) to study the deeper relationships among the GRAS family members in cotton. On the basis of previous GRAS family studies [8], we divided all GRAS members identified into 15 subfamilies: SCR, DLT, OS19, LAS, SCL4/7, OS4, OS43, DELLA, PAT1, SHR, HAM, SCL3, LISCL, G_GRAS and PSG. G_GRAS is cotton-special subfamily, and PSG only contains genes of *P. patens* and *S. moellendorffii*. Most of the GRAS members enrich in LISCL, SHR, PAT1 and HAM. The distribution of genes of *G. hirsutum* among different subfamilies was as following: SCR (10), DLT (4), OS19 (4), LAS (2), SCL4/7 (4), OS4 (4), OS43 (2), DELLA (8), PAT1 (27), SHR (28), HAM (24), SCL3 (9), LISCL (20) and G_GRAS (4). To check the results obtained by the NJ method, we constructed phylogenetic trees using the UPGMA evolution methods (Additional file 2: Figure S1). The final trees constructed with the two methods were almost same except two clades marked with imaginary lines (Additional file 2: Figure S1). Here, we identified GRAS members in moss (*P. patens*), fern (*S. moellendorffii*), dicotyledon (*G. hirsutum, G. raimondii, G. arboreum and A. thaliana*) and monocotyledon (*O. sativa*), finding there were GRAS members of moss and fern in every subfamily. This suggests that GRAS subfamilies occurred before these species. We could not, however, identified any GRAS members in almost all algaes, indicating that GRAS family may produce by bacterial infection [7] in the period between algae and moss. Finally, we build a model to describe the possible evolution process (Additional file 3: Figure S6).

### Chromosomal locations and duplication of GRAS members

Analysis of the *G. hirsutum* genome sequence with the GFF3 profile revealed the chromosomal position of each GRAS member. As a result, 141 GRAS transcription factor genes were mapped onto the *G. hirsutum* chromosomes named according to their chromosomal order, A01–A13 and D01–D13. However, 9 GRAS members were not obviously mapped to any chromosome and were located on unattributed scaffolds (Additional file 1: Table S1). It indicates that genes’ distribution on A chromosomes was almost same with the situation D chromosomes (Additional file 4: Figure S2).

**Fig. 2:**
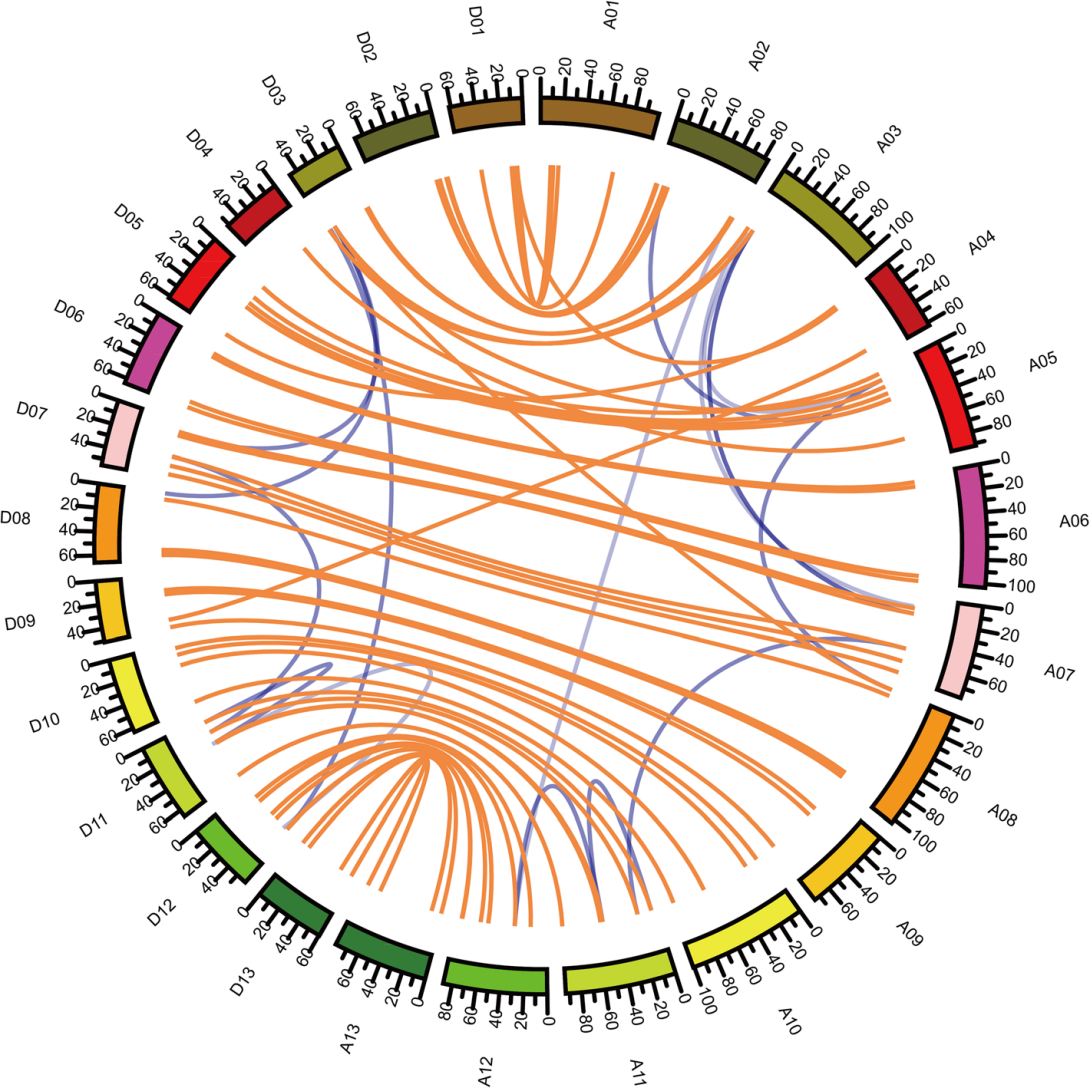
Duplication events among GRAS members in *G. hirsutum* L.. Yellow lines indicate homologous genes between A and D chromosomes, and blue lines indicate segmental duplications between A or D chromosomes

Gene duplication analysis revealed that five kinds of duplication events (singleton, dispersed, proximal, tandem and segmental) (Additional file 5: Table S4) were reasons for GRAS family expansion in the cotton genome. We identified 11 genes belonging to tandem duplications, 41 genes belonging to singleton duplications, 21 genes belonging to dispersed duplications, 4 genes belonging to proximal duplications and 64 genes belonging to segmental duplications, which showed that segmental duplication events were the main reason for GRAS members’ evolution in *G. hirsutum.* In addition, homologous gene among GRAS members and segmental genes’ pairs were showed by marking with yellow and blue lines respectively (Fig. 2). And we also calculated the Ka and Ks of segmental genes’ pairs, finding Ka/Ks were all lower than 1, which indicated that purified selection happened on these genes. Finally, we identified 6 pairs of tandem duplicated gene pairs (Additional file 5: Table S4).

### Exon-intron structure and domain architecture of GRAS members

**Fig. 3:**
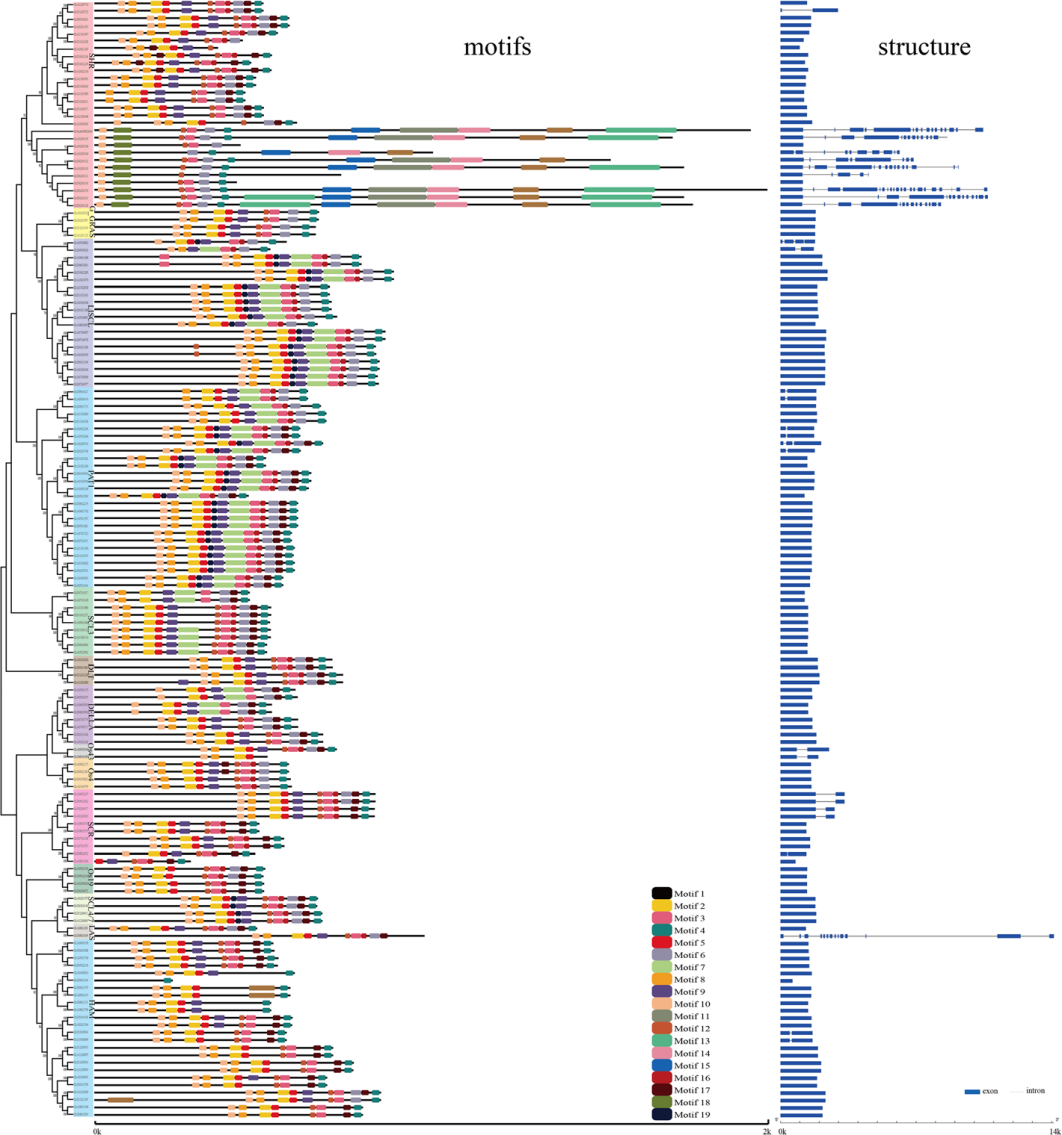
Phylogenetic relationship, motifs and gene structures of GRAS members in *G. hirsutum* L.

Using the online software Gene Structure Display Server (GSDS 2.0, http://gsds.cbi.pku.edu.cn/index.php), we generated a graph to show the exon-intron structures and GRAS domain positions of every GRAS member (Fig. 3c). And it clearly shows there was a conserved motif in the same subfamily. And the *p-values* of motifs showed on every protein were lower than 1e^− 5^ (Fig. 3b). Besides, almost 30% members containing introns belong to SHR. And the related motifs were also different with others, whatever the length and motif orders. All motifs’ logo were also showed (Additional file 6: Figure S4). We also could find that the members cotaining many introns lack the motif 17, which is very conservative in other proteins. Several special motifs (motif 11, motif 15 and motif 18) arise in these proteins.

**Fig. 4:**
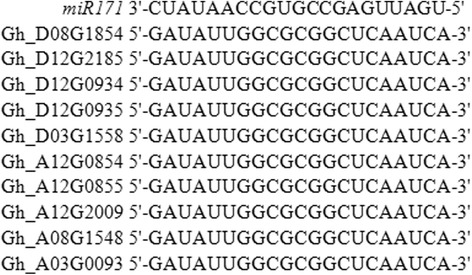
Potential GRAS members targeted by gh-miRNA 171 in *G. hirsutum* L.

According to previous studies, some GRAS members are regulated by microRNA 171 [32]. Thus, we queried the previously reported binding site sequence targeted by gh-microRNA 171 in cotton [33] for every member of the GRAS family. The results indicated that only 10 genes from the HAM subfamily (Fig. 4) were unambiguously complementary to the gh-microRNA 171 sequence.

### Expression profiles of GRAS members under abiotic stresses, among different tissues and development stages

**Fig. 5:**
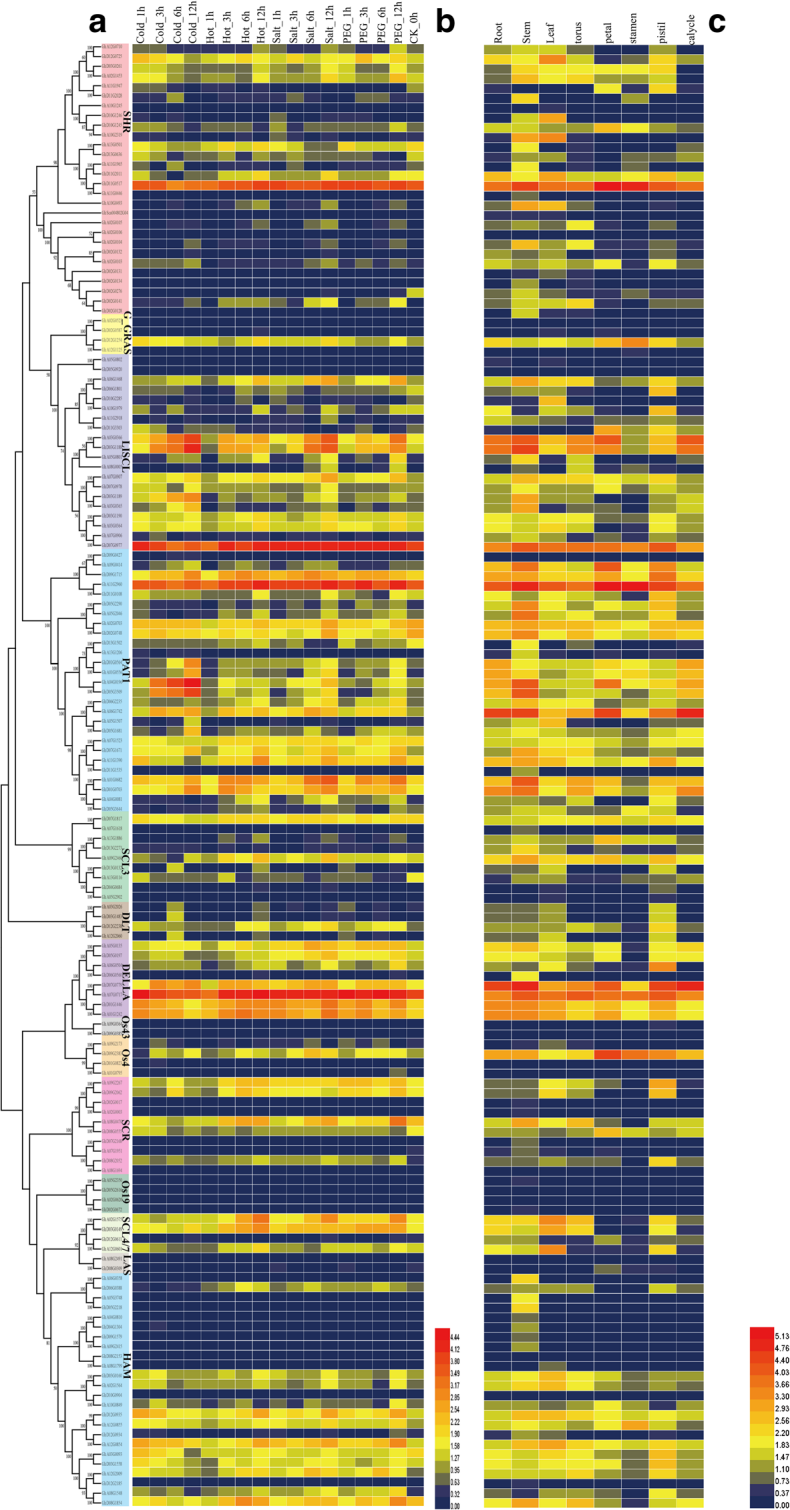
Gene expression profiles of GRAS members in *G. hirsutum* L.. **a** A tree of the GRAS members in *G. hirsutum* L. **b** Gene expression profiles of GRAS members under different abiotic stresses. **c** Gene expression profiles of GRAS members in different tissues

To investigate the expression profiles of the *G. hirsutum* GRAS members under abiotic stresses (heat, cold, salt and drought), we used publicly available transcriptome data [34] . Among several subfamilies (LISCL, HAM, PAT1 DELLA, SCL4/7 and SCR), genes had higher expression levels under stresses comparing with CK (Fig. 5b). In order to show the variation trend directly, several broken line graphs were built (Additional file 7: Figure S7). Additionally, we found that many genes were indeed induced by stress treatments (Additional file 7: Figure S7). Besides, in order to study the possible reasons of stress resistance, we also analyzed the expression pattern in different tissues (Fig. 5c) and development stages (Additional file 8: Figure S5). Most of *G. hirsutum* GRAS members have higher expression level in root, stem, leaf and pistil (Fig. 5b). Besides, about 70% genes in LISCL and PAT1 have higher expression level in different development stages (Additional file 8: Figure S5), suggesting these members maybe house-keeping genes.

### qRT-PCR on 15 GRAS members under abiotic stresses

**Fig. 6:**
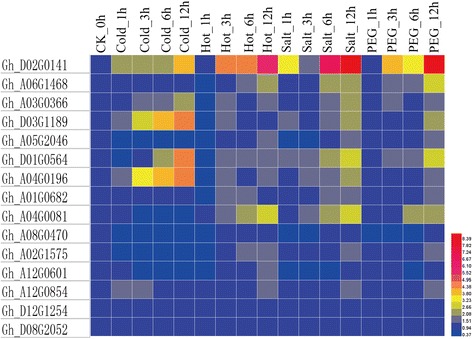
The examination results of 15 selected GRAS members via qRT-PCR

To validate the publicly available data downloaded from NCBI, 15 GRAS members whose expression level were up-regulated under stress treatments. We designed primers for 15 selected genes (Additional file 9: Table S2) and conducted a qRT-PCR experiment. According to the results, the expression profiles (Additional file 10: Table S5) of most selected genes were basically consistent with the published data, indicating the FPKM values (Additional file 11: Table S3) was reliable (Fig. 6). As the situation shown, *Gh_D02G0141, Gh_A06G1468, Gh_A03G0366, Gh_D03G1189, Gh_A05G2046, Gh_D01G0564, Gh_A04G0196, Gh_A01G0682* and *Gh_A04G0081* were significantly up-regulated under salt and PEG stresses (*P < 0.01*). What’s more, *Gh_D02G0141* and *Gh_A04G0081* were all significantly up-regulated under hot (*P < 0.01*), which is same as the phenomenon shown by Zhang [35] (Fig. 5b). As for cold, *Gh_D02G0141, Gh_A03G0366, Gh_D03G1189, Gh_D01G0564* and *Gh_A04G0196* were up-regulated (*P < 0.01*). What valuable is that *Gh_D02G0141* have obvious resistance against four abiotic stresses, suggesting that this gene should work as the candidate for breeding stress-resistance cotton.

## Discussion

GRAS family members have diverse functions and play important roles in plant development and physiological processes, such as GA signal transduction, root development, axillary shoot development, and transcriptional regulation in response to abiotic and biotic stresses [35]. To date, many studies have been undertaken to elucidate the evolutionary history of the GRAS family in a number of plant species, including *A. thaliana* [9], *O. sativa* [9], *Castor Bean* [8], *tobacco* [10], *Populus* [11], *Chinese cabbage* [12], *Grapevine* [13] and *Jatropha curcas* [36]. Nevertheless, few studies have been conducted in cotton. In this study, we performed a comprehensive analysis of the GRAS members in cotton, including their phylogenetic relationships, gene structures, conserved motifs, chromosomal locations, duplication events, and expression profiles under abiotic stresses.

### Phylogeny, exon-intron structures and expansion of GRAS member in cotton

In this study, we divided the GRAS members identified in *O. sativa, A. thaliana*, *P. patens*, *S. moellendorffii*, and cotton (*G. hirsutum, G. arboreum and G. raimondii*) into 15 subfamilies. According to our results, most GRAS members (82%) have no intron. Interestingly, many SHR subfamily members blended with introns nabbed in *G. hirsutum*, suggesting these genes may be more recently evolved GRAS members in cotton. In order to explain this hypothesis, we searched the GRAS members range from algae to monocotyledon. We did not find any GRAS members in several kinds of algae (Additional file 3: Figure S6). However, there were many GRAS members in other plants. In considration previous report on deriving from bacteria [7], we speculated that the origin of GRAS family may happen by the infection of bacteria in stage between algae and moss. What’s more, we find there were many introns in *P. patens* and cotton, mainly enriching in SHR subfamily (Additional file 12: Figure S3). There existed, however, few intron among other species or subfamilies. According to previous reports, introns may play a role in the evolution of plants [37]. In the earlier stage of expansion, there always raised a lot of introns, losing slowly as time went by [38]. So, the more advanced of the specie, the fewer intron among genome [39] . Sometimes, however, introns also could become larger in the process of evolution, whose reason maybe the necessarity for new function [40] . Besides, tandem duplication always resulted in the increasing of introns, leading to the generation of new genes [41]. In this study, cotton’s introns increased which mainly belong to SHR, suggesting these genes may arise before the differentiation of monocotyledon and dicotyledon. What’s more, 50% tandem duplicated gene pairs (Gh_A02G0103, Gh_A02G0104; Gh_A02G0105, Gh_A02G0106; and Gh_A02G0104, Gh_A02G0105) belong to SHR subfamily and are located in chromosome A02, indicating tandem duplication lead to the increasing of introns and expansion of genes. Furthermore, we can speculate the different motif among these genes that have many introns may possess special function for cotton’s growth and development, which need deeper researches.

### The model for the evolution of GRAS family

Additionally, we proposed a model for the evolution of this gene family (Additional file 3: Figure S6). Subfamilies including SCR, DLT, Os19, LAS, SCL4/7, Os43, DELLA, PAT1, SHR, HAM, SCL3, LISCL, G_GRAS and PSG origin after the alga species. And then, Os4 was origin after the bryophyte. PSG, however, lost after the fern. What’s more, some families including Os4, Os43 and G_GRAS loss in some dicots (such as *A. thaliana*). And subfamily G_GRAS lost after the dicots. What mentioned above shows that most subfamilies origin in the beginning. It also indicated that G_GRAS genes may have a special effect on the growth and development of cotton.

### Analysis of the possible functions of the GRAS members

Importantly, the DELLA motif is required for the GA-induced degradation of DELLA proteins [42, 43]. Here eight DELLA genes were identified (Gh_A01G1242, Gh_A05G0135, Gh_A06G0504, Gh_A07G0717, Gh_D01G1446, Gh_D05G0197, Gh_D06G0388 and Gh_D06G2235), which possess DELLA domain and play important roles in regulation of the elongation of roots. In addition, previous reports have shown that microRNA 171 can regulate the expression of some GRAS members [44] Overexpressing miR171 had effect on plant height, flowering time, leaf architecture, phase transitions and floral meristem determinacy [45]. In this study, to our surprise, 10 GRAS members belonging to the HAM subfamily contained potential target sites for *gh-miRNA 171* binding, indicating a possible regulatory mechanism that needs to be studied in detail. Additionally, about 50% HAM subfamily members had higher expression levels under various abiotic stresses and among tissues (Fig. 5), suggesting that *gh-miRNA 171* may regulate pleiotropic phenotypes. So, this result would be helpful for us to study its signal path on abiotic stresses or generation.

### Expression profiles of GRAS members in cotton

Many reports have provided evidence that GRAS members are important for plants to regulate their biochemical activities under various abiotic stresses [46]. According to data from a previous study [34], about 91% of the GRAS members identified in *G. hirsutum* are affected by a variety of stress conditions. Previous studies has shown that *NtGRAS1* [47] and *AtSCL14* were stress-induced members which belonged LISCL subfamily. Here, we found that *Gh_D02G0141, Gh_A03G0366, Gh_D03G1189, Gh_D01G0564* and *Gh_A04G0196* were obviously up-regulated when being under the cold, salt and PEG treatments. The expression level of *Gh_A04G0081*, did not change under cold, but be up-regulated under other three kinds of stressess. In addition, *Gh_D01G0564* and *Gh_A04G0196* belonging to PAT1 subfamily were up-regulated (*P < 0.01*) whatever the stresses, inducating this gene may play important roles in anti-stress for cotton, which show no difference with the report on *VaPAT1* [27]. Over-expression of *Gh_GRAS42* may improve cotton’s tolerance to abiotic stresses. What’s more, Ma [25] has proved that *PeSCL7*, the homologous gene of *AtSCL7* of SCL4/7 subfamily member, could be induced by salt and drought stresses, which was helpful for *Arabidopsis thaliana* to improve salt and drougt tolerance. We also found that SCL4/7 subfamily member *Gh_A02G1575* was up-regulated (*P < 0.01*) when treated under PEG and salt (Fig. 5b), which show it is important for cotton to resist abiotic stresses. What’s more, its expression level was higher among root, stem, leaf and pistil, suggesting it may also influenced development and generation (Fig. 5c). Interestingly, *Gh_ D02G0141,* whose expression level was clearly high under all kinds of stresses (*P < 0.01*), indicating that this gene may be a good gene for breeding cotton resisting abiotic stresses. In addition, 50–70% genes of LISCL, PAT1 and DELLA have continual higher expression level in different development stages, showing these genes maybe house keeping genes (Additional file 8: Figure S5B). Together, our results show that *G. hirsutum* GRAS members are necessary for abiotic stress resistance and could be used to improve the stress tolerance of cotton in future studies.

## Conclusions

In this study, 150 GRAS members in *G. hirsutum*, 77 GRAS members in *G. arboreum*, 82 GRAS members in *G. raimondii*, 33 GRAS members in *A. thaliana* [9] 42 GRAS members in *P. patens*, *46* GRAS members in *S. moellendorffii*, and 50 GRAS members in *O. sativa* [8] were identified. The GRAS family was divided into 5 subgroups based on the phylogenetic tree and the distribution of conserved motifs. Intron/exon structure and motif analysis showed GRAS members may produce some genes cotaining introns, whose expansion may because tandem duplication. The chromosomal location of every GRAS member was identified, and duplication analysis showed that segmental duplication events played the main role in GRAS family expansion. And purified selection worked on these duplicated pairs. Additionly, we also found that *gh-miRNA 171*, which plays a regulatory role in GRAS member expression according to previous reports, had target genes in *G. hirsutum*, all of which belonged to the HAM subgroup. Finally, the expression patterns of the GRAS members under abiotic stresses and among tissues or development stages were explored using transcriptome data and qRT-PCR. In conclusion, our work provides functional insights into the roles of GRAS members in the resistance of cotton to abiotic stresses.

## Methods

### Identification of GRAS family members in cotton and other species

We downloaded the latest versions of the genome annotations for the following species from the Phytozome v11 database (https://phytozome.jgi.doe.gov/pz/portal.html): *C. reinhardtii* (V5.5), *P. patens* (V3.3), *S. moellendorffii* (V1.0), *A. thaliana* (TAIR annotation release 10) and *O. sativa* (MSU annotation release 7.0). Additionally, we downloaded the *G. hirsutum* [34] (https://www.cottongen.org/species/Gossypium_hirsutum/nbi-AD1_genome_v1.1), *Gossypium arboreum* [48] (https://www.cottongen.org/species/Gossypium_arboreum/bgi-A2_genome_v2.0) and *Gossypium raimondii* [49] (https://www.cottongen.org/species/Gossypium_raimondii/jgi_genome_221) genome sequences from COTTONGEN (https://www.cottongen.org/). We download the hidden Markov model (HMM) profile (PF03514.13) [11]. The HMM profile was then used to perform HMM searches against annotated protein databases from different genomes with an E-value cutoff of 1e^− 5^ in the program HMMER 3.1b2 [50]. To confirm that the predicted proteins contained GRAS domains, we evaluated them using the SMART software [51] (http://smart.embl-heidelberg.de/). Proteins without GRAS domain or the length were shorter than half of the typical GRAS domain length (350aa) [46]. And at the same time, we also recorded the GRAS domain positions so that we could retrieve the domain sequences for next study. What’s more, we arranged GRAS proteins’s pIs, lengths, weights, chrs, related genes’ positions and subfamilies.

### Phylogenetic analysis

The GRAS domain amino acid sequences were determined using the complete GRAS protein sequences identified by HMM profile searches and the domain positions identified by the SMART software. The GRAS proteins’ domain sequences in *A. thaliana*, *O. sativa, C. reinhardtii*, *P. patens*, *S. moellendorffii* and cotton were aligned using muscle method of MEGA6.0 with the default parameters. The aligned sequences were then subjected to phylogenetic analysis with the neighbor joining (NJ) method. The consensus tree was built using MEGA 6.0 [52] under the P-distance model and the pairwise deletion option with 1000 bootstrap replicates. Additionally, we used the UPGMA method to examine the results from the NJ method. Finally, we divided subfmily according previous reports and modified the phylogenetic trees by AI software. PoGOs were defined as described in Del Bem and Vincentz [53].

### Analysis of the conserved motifs and gene structures

The online software Gene Structure Display Server (GSDS 2.0) (http://gsds.cbi.pku.edu.cn/index.php) [54] was used to show the gene structures (introns and exons) by submitting the related profile extracted from the genome’s GFF3 profile (ftp://ftp.bioinfo.wsu.edu/species/gossypium_hirsutum/NAU-NBI_G.hirsutum). Then we performed motifs’ analysis by local MEME software with following parameters: minsites 6, maxsites 149, minw 6, maxw 200 and nmotifs 19.

### Chromosomal locations analysis of GRAS members

The chromosomal positions of all GRAS members were confirmed using the cotton genome annotation file (ftp://ftp.bioinfo.wsu.edu/species/Gossypium_hirsutum/NAU-NBI_G). The length of each chromosome was calculated using a Perl script. Then, we made a diagram of the GRAS members that showed duplications among 26 chromosomes and identified five types of gene duplication events with MCScanX using the GRAS protein sequences and the position data in chromosomes [55]. Additionally, we made a diagram of the chromosomal locations with the MapInspect software [56].

### Calculation of Ka/Ks values

The protein sequences from segmentally duplicated pairs and orthologous pairs were aligned using Clustal X 2.0. Then, the aligned sequences were converted to the original cDNA sequences by the PAL2NAL program [57] (http://www.bork.embl.de/pal2nal/). Finally, The CODEML program of the PAML package [58] was used to estimate the synonymous (Ks) and nonsynonymous (Ka) substitution rates.

### Expression analysis of GRAS members

The expression levels of GRAS members were measured using the RNA-sequencing data of *G. hirsutum* TM-1 among different tissues, development stages and stresses (cold, heat, salt and drought), which were downloaded from the NCBI Sequence Read Archive (SRA: PRJNA248163) (http://www.ncbi.nlm.nih.gov/bioproject/PRJNA248163/) [59] The FPKM values (Additional file 11: Table S3) were performed treament of ln(X + 1), whose results were than used for building heatmap. Finally, a heatmap of GRAS expression was produced using the HemI 1.0 software [60].

### RNA isolation and the qRT-PCR analysis

In order to validate the expression data we downloaded and prepare for further study, we selected 15 genes whose expression levels were obviously higher under stresses. The qRT-PCR primers were designed (Additional file 9: Table S2) and seedlings of CCRI24 was used. After 4 weeks of cultivation, plants at the three-leaf stage were subjected to four kinds of abiotic stress. The treatments were as follows: cold (4 °C for 1,3,6,12 h), heat (38 °C for 1,3,6,12 h), salt (400 mM NaCl for 1,3,6,12 h), and drought (20% PEG for 1,3,6,12 h) [34]. Samples collected at 0 h were regarded as controls. Ten plants were included in each treatment group and their leaves were collected at the three-leaf stage.

RNA was extracted from the detached leaves using an RNA Aprep Pure Plant Kit (TIANGEN, Beijing, China). The quality and concentration of each RNA sample was determined using gel electrophoresis and a NanoDrop 2000 spectrophotometer (only RNA that met the criteria A260/280 ratio = 1.8–2.1 and A260/230 ratio ≥ 2.0 was used for further analyses and stored at − 80 °C). The high-quality RNA samples were treated with DNase I (TaKaRa, TOYOBO, Japan) to eliminate contaminating genomic DNA. cDNA was synthesized from 1 μg of RNA in a 20 μL reaction volume using the PrimeScript™ RT Reagent Kit (Takara, Dalian, China) according to the manufacturer’s manual. qRT-PCR experiments were conducted to measure the expression levels of the GRAS members under stress treatments. qRT-PCR analysis was performed using the Applied Biosystems 7500 Real-Time PCR system and the SYBR premix Ex Taq™ kit (TaKaRa, Japan). Target gene amplification was checked by the SYBR Green fluorescence signal. The cotton constitutive His gene [61] (GenBank accession no. AF024716) was used as a reference gene and 15 pairs of specific GRAS primers were used for qRT-PCR. Three biological replicates were included for each treatment, each consisting of three technical replicates.. The following thermal circulation conditions were used: 95 °C for 30 s, followed by 40 cycles of 95 °C for 5 s and 60 °C for 1 min. Expression levels were calculated as the mean signal intensity across the three replicates. Following the PCR, a melting curve analysis was performed. Ct or threshold cycle values were used for relative quantification of the input target number. The relative fold difference (N) is the number of treated target gene transcript copies relative to the untreated gene transcript copies, and was calculated according to Livak and Schmittgen (2001) as follows: *N* = 2^-ΔΔCt^ = 2^-(ΔCt treated − ΔCt control)^, where ΔΔCt = ΔCt of the treated sample minus ΔCt of the untreated control sample. Student’s t-test were conducted in order to study whether the variation of expression level under CK and treatments was meaningful.

## Additional files

Additional file 1: **Table S1.** GRAS members identified in *O. sativa*, *P. patens*, *S. moellendorffii*, *A. thaliana*, *G. hirsutum*, *G. arboreum* and *G. raimondii*. (XLS 111 kb)

Additional file 2: **Figure S1.** Phylogenetic tree built with the UPGMA method. (TIF 8356 kb)

Additional file 3: **Figure S6.** Phylogenetic trees and evolutionary profile of GRAS genes in green plants. (A) Phylogenetic tree showing the evolutionary relationship between plants (marked with different colors) GRASs. Tree topology is a consensus from NJ, UPGMA. Bootstrap values from the original trees higher than 50% are shown (NJ/UPGMA). (B) Evolutionary profile of GRAS subfamilyes’ genes in green plants. (TIF 1487 kb)

Additional file 4: **Figure S2.** Chromosomal locations of GRAS members in *G. hirsutum* L.. (TIF 2000 kb)

Additional file 5: **Table S4.** The duplication types of GRAS members in *Gossypium hirsutum* L. and segmental gene pairs with their Ka/Ks values. Besides, six tandem duplicated gene pairs were also performed. (XLSX 14 kb)

Additional file 6: **Figure S4.** Motifs describution of GRAS members in *G. hirsutum* L.. development stages in *G. hirsutum* L.. (TIF 6550 kb)

Additional file 7: **Figure S7.** The expression level of different GRAS genes under various stress. (TIF 1752 kb)

Additional file 8: **Figure S5.** Gene expression profile of GRAS members among different. (TIF 1197 kb)

Additional file 9: **Table S2.** Premiers of GRAS members selected for qRT-PCR. (XLS 22 kb)

Additional file 10: **Table S5.** The expression data of qRT-PCR. (XLS 36 kb)

Additional file 11: **Table S3.** The public available expression profile of GRAS members. (XLS 123 kb)

Additional file 12: **Figure S3.** The exon-intron structure of every GRAS member in six species. (A) *P. patens* (B) *O. sativa* (C) *S. moellendorffii* (D) *G. arboreum* (E) *A. thaliana* (F) *G. raimondii*. (TIF 1283 kb)

## Abbreviations

D: Dalton
FPKM: Fragments Per Kilobase of exon model per Million mapped reads
G_GRAS: Gossypium GRAS members
GA: Gibberellic acid
GRAS: GAI, RGA, and SCR
GSDS: Gene structure display server
HMM: Hidden markov model
MW: Molecular weight
NJ: Neighbor joining
NLS: Nuclear localization signals
PEG: Polyethylene glycol
pI: Isoelectric point
PSG: *P. patens* and *S. moellendorffii GRAS members*
qRT-PCR: Quantitative reverse transcription-polymerase chain reaction
