# Supplementary figures and images for: Genome-wide analysis of GRAS transcription factor gene family in Gossypium hirsutum L
Source: BMC Genomics. 2018 May 9;19:348. doi: 10.1186/s12864-018-4722-x (PMC5944045; doi:10.1186/s12864-018-4722-x)

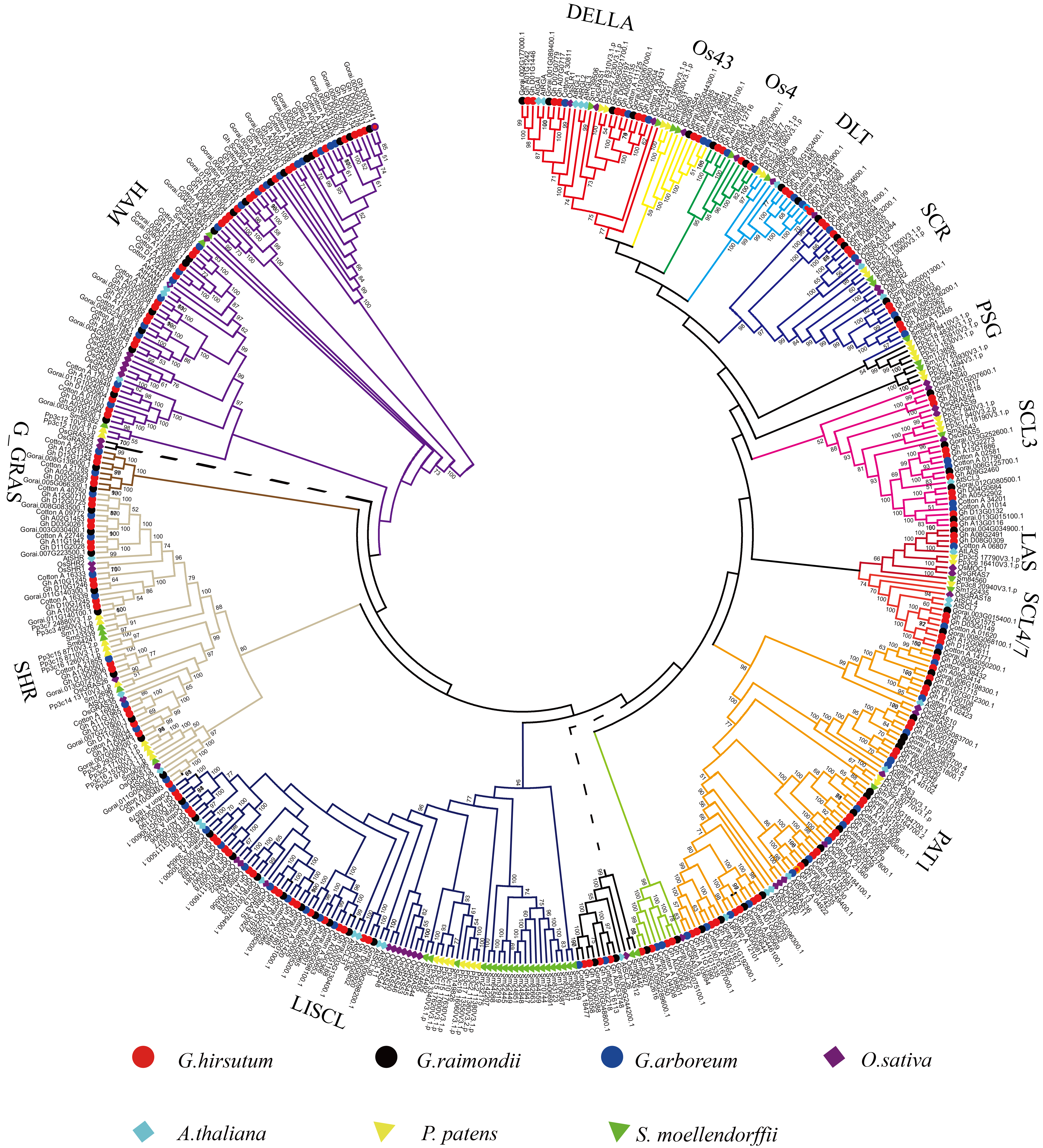

Supplement: Supplementary file 2 — Figure S1. Phylogenetic tree built with the UPGMA method. (TIF 8356 kb) [file 12864_2018_4722_MOESM2_ESM.tif]

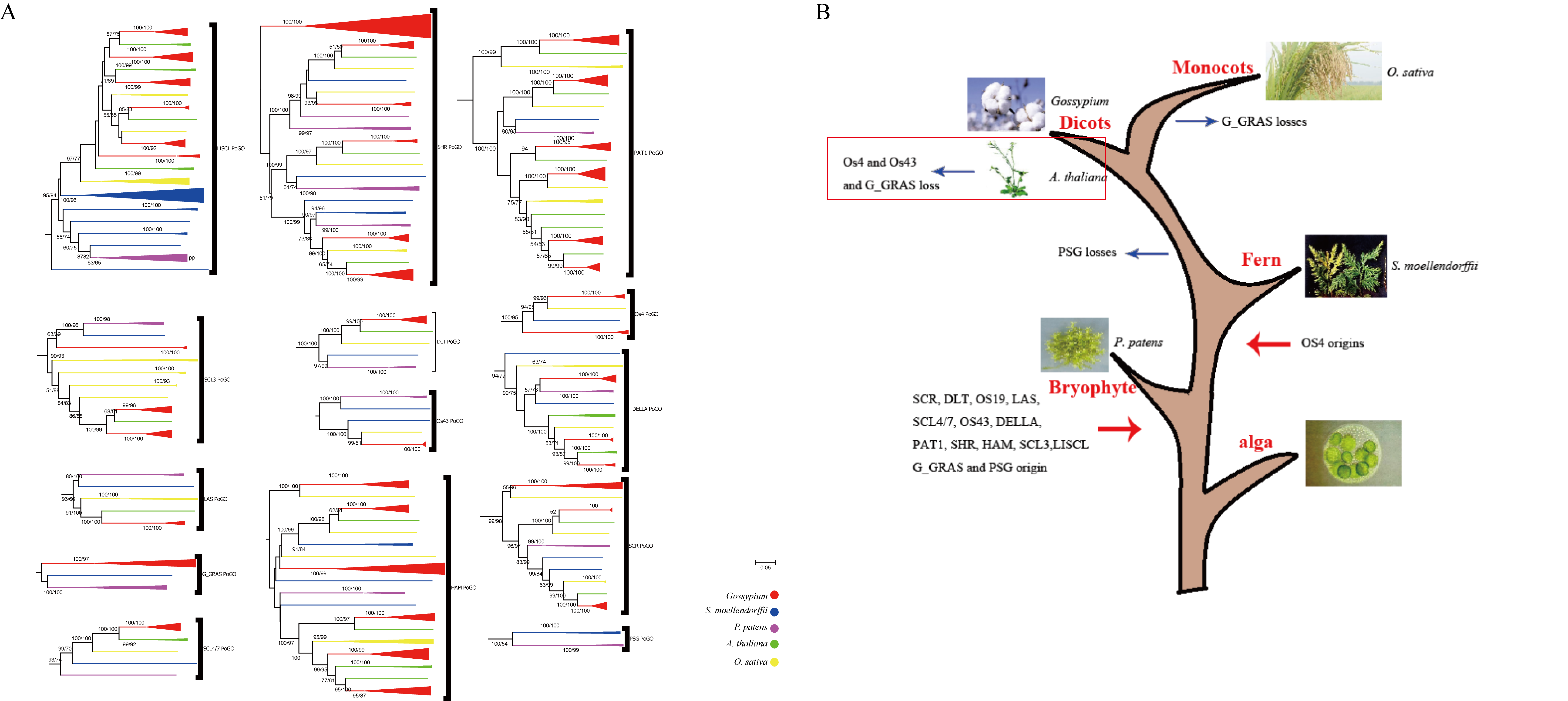

Supplement: Supplementary file 3 — Figure S6. Phylogenetic trees and evolutionary profile of GRAS genes in green plants. (A) Phylogenetic tree showing the evolutionary relationship between plants (marked with different colors) GRASs. Tree topology is a consensus from NJ, UPGMA. Bootstrap values from the original trees higher than 50% are shown (NJ/UPGMA). (B) Evolutionary profile of GRAS subfamilyes’ genes in green plants. (TIF 1487 kb) [file 12864_2018_4722_MOESM3_ESM.tif]

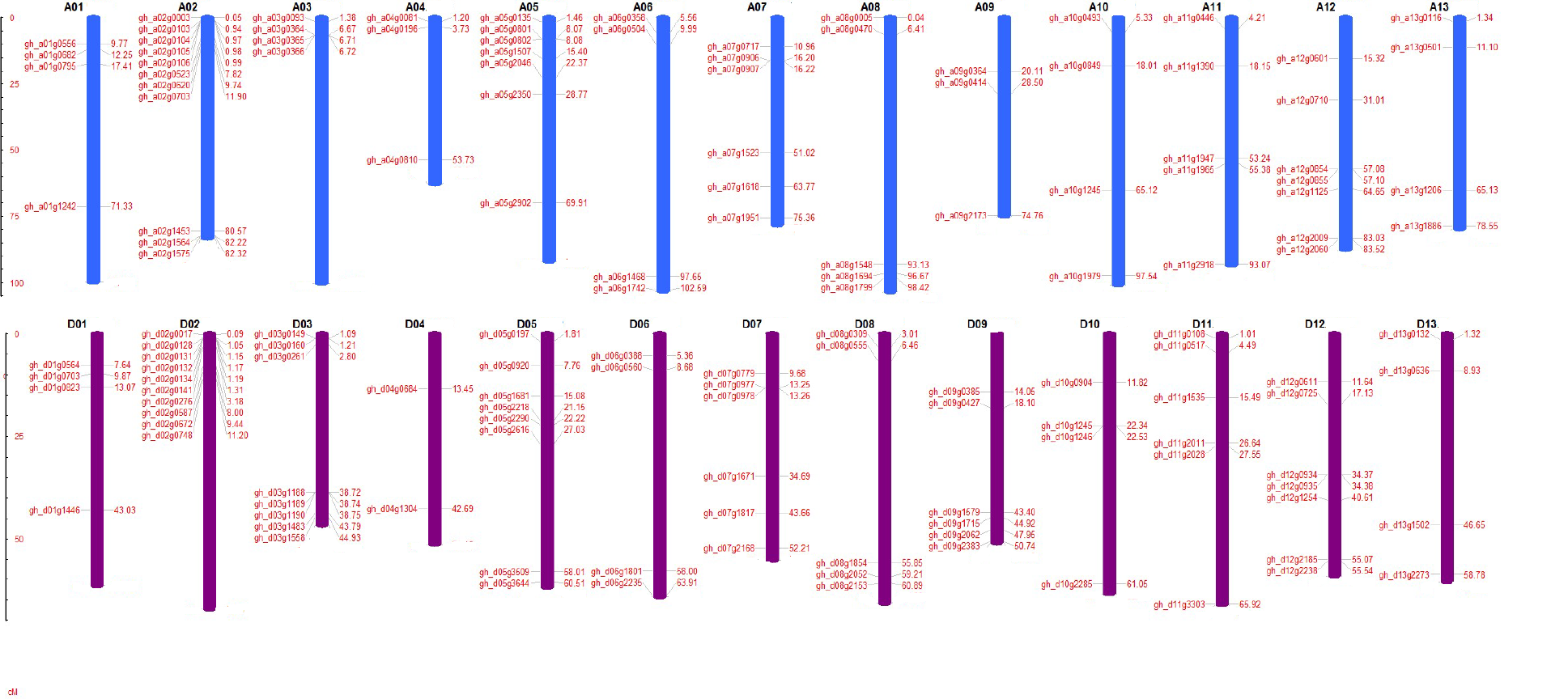

Supplement: Supplementary file 4 — Figure S2. Chromosomal locations of GRAS members in G. hirsutum L.. (TIF 2000 kb) [file 12864_2018_4722_MOESM4_ESM.tif]

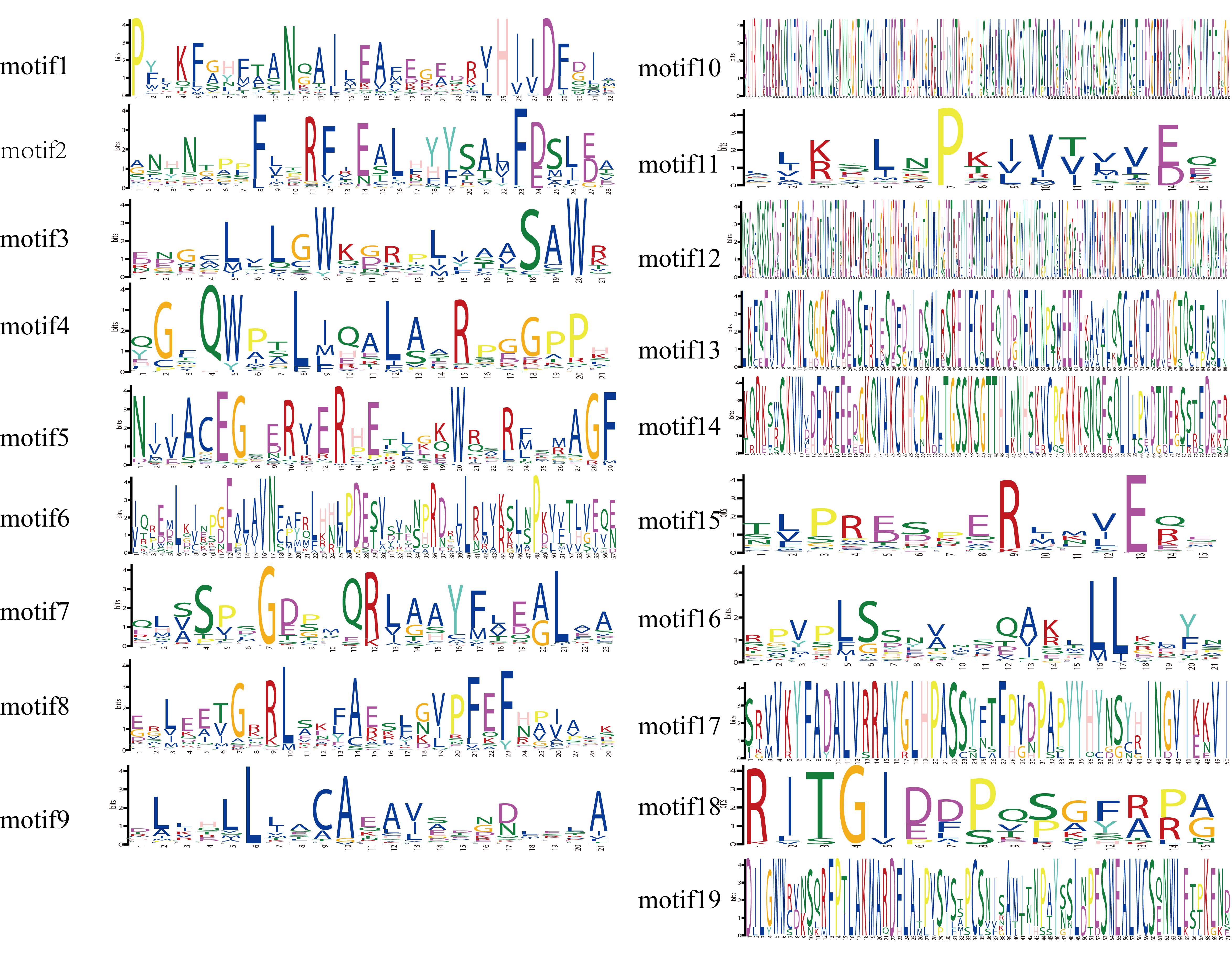

Supplement: Supplementary file 6 — Figure S4. Motifs describution of GRAS members in G. hirsutum L.. development stages in G. hirsutum L.. (TIF 6550 kb) [file 12864_2018_4722_MOESM6_ESM.tif]

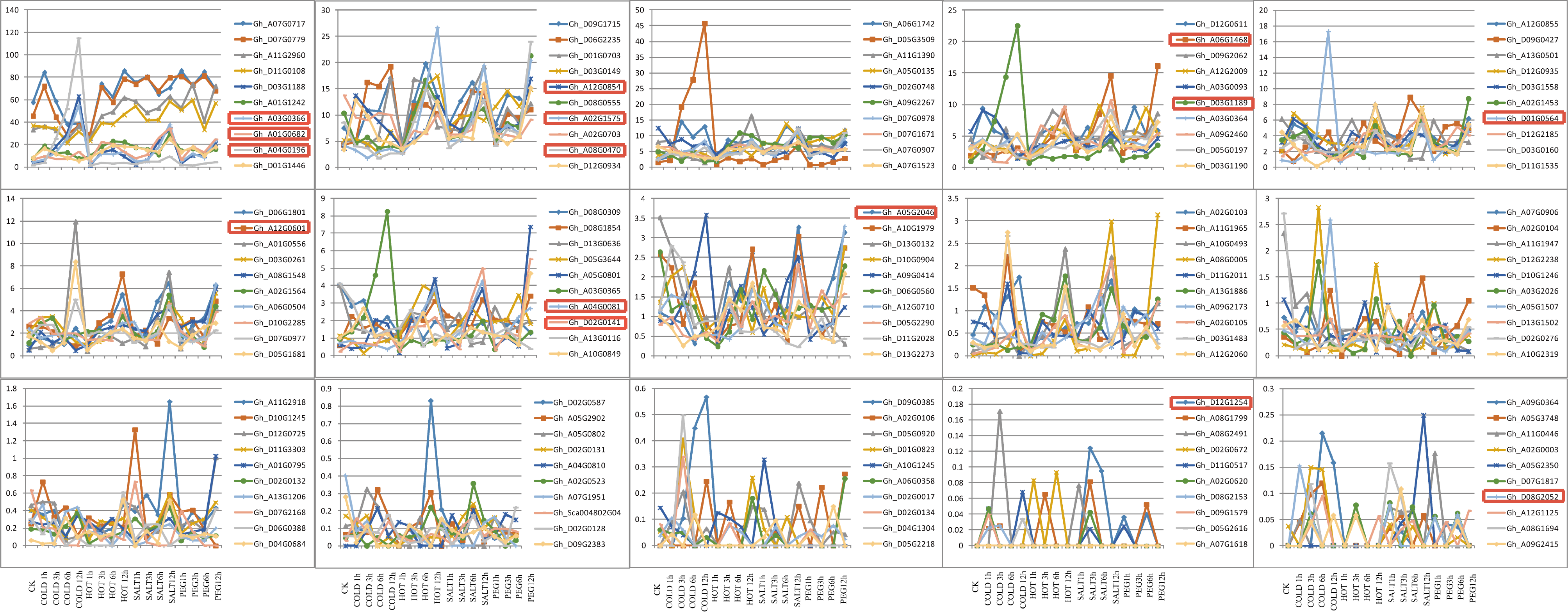

Supplement: Supplementary file 7 — Figure S7. The expression level of different GRAS genes under various stress. (TIF 1752 kb) [file 12864_2018_4722_MOESM7_ESM.tif]

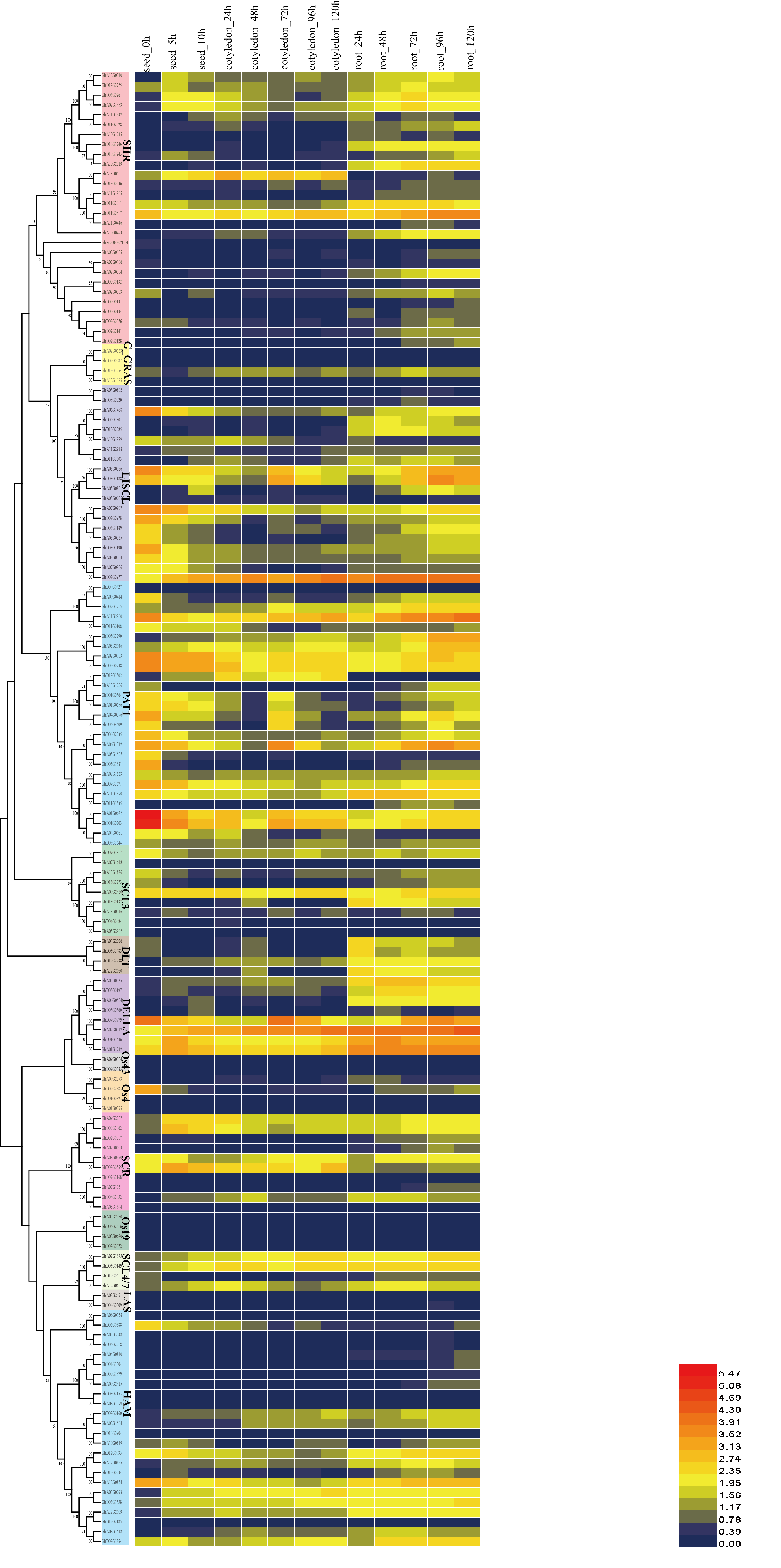

Supplement: Supplementary file 8 — Figure S5. Gene expression profile of GRAS members among different. (TIF 1197 kb) [file 12864_2018_4722_MOESM8_ESM.tif]

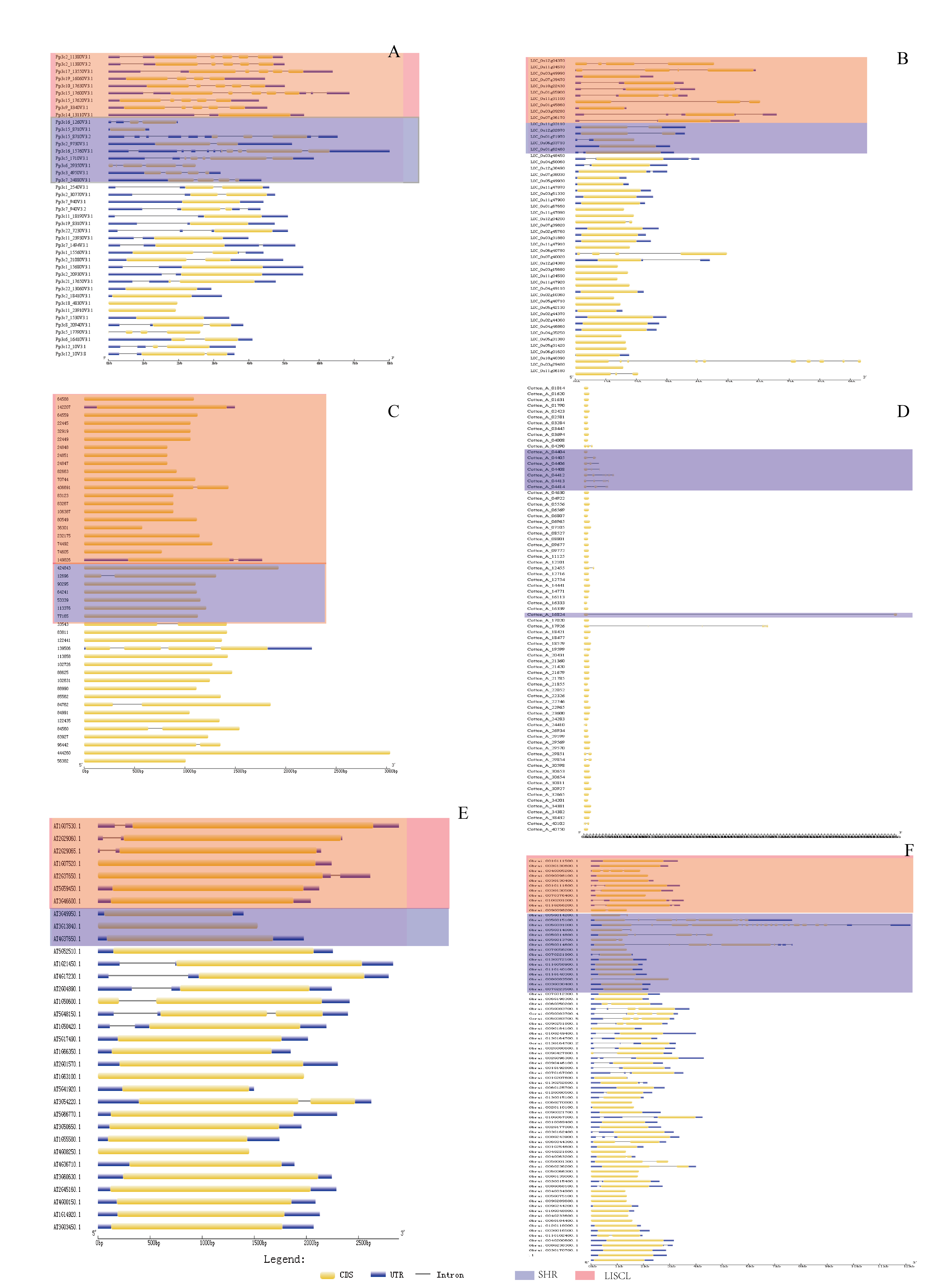

Supplement: Supplementary file 12 — Figure S3. The exon-intron structure of every GRAS member in six species. (A) P. patens (B) O. sativa (C) S. moellendorffii (D) G. arboreum (E) A. thaliana (F) G. raimondii. (TIF 1283 kb) [file 12864_2018_4722_MOESM12_ESM.tif]
